# Serum Carotenoids Reveal Poor Fruit and Vegetable Intake among Schoolchildren in Burkina Faso

**DOI:** 10.3390/nu10101422

**Published:** 2018-10-04

**Authors:** Jean Fidèle Bationo, Augustin N. Zeba, Souheila Abbeddou, Nadine D. Coulibaly, Olivier O. Sombier, Jesse Sheftel, Imael Henri Nestor Bassole, Nicolas Barro, Jean Bosco Ouedraogo, Sherry A. Tanumihardjo

**Affiliations:** 1 Centre Muraz, Post Office Box 390, Bobo Dioulasso 01, Burkina Faso; jeanfidelebationo@gmail.com; 2 Institute de Recherche en Sciences de la Santé, Post Office Box 545, Bobo Dioulasso 01, Burkina Faso; nawidzeba@gmail.com (A.N.Z.); nadineyd@yahoo.fr (N.D.C.); sombieolivier@yahoo.fr (O.O.S.); jbouedraogo@gmail.com (J.B.O.); 3 University of Ghent, Department Public Health; 9000 Ghent, Belgium; Souheila.Abbeddou@ugent.be; 4 University of Wisconsin-Madison, Nutritional Sciences Department; Madison, WI 53706, USA; jsheftel@wisc.edu; 5 Université Ouaga 1 Joseph Ki-Zerbo, Ouagadougou 03, Burkina Faso; ismael.bassole@gmail.com (I.H.N.B.); barronicolas@yahoo.fr (N.B.)

**Keywords:** α-carotene, β-carotene, β-cryptoxanthin, carotenoids, lutein, provitamin A, retinol, vitamin A

## Abstract

The health benefits of fruits and vegetables are well-documented. Those rich in provitamin A carotenoids are good sources of vitamin A. This cross-sectional study indirectly assessed fruit and vegetable intakes using serum carotenoids in 193 schoolchildren aged 7 to 12 years in the Western part of Burkina Faso. The mean total serum carotenoid concentration was 0.23 ± 0.29 µmol/L, which included α- and β-carotene, lutein, and β-cryptoxanthin, and determined with serum retinol concentrations in a single analysis with high performance liquid chromatography. Serum retinol concentration was 0.80 ± 0.35 µmol/L with 46% of children (*n* = 88) having low values <0.7 µmol/L. Total serum carotene (the sum of α- and β-carotene) concentration was 0.13 ± 0.24 µmol/L, well below the reference range of 0.9–3.7 µmol carotene/L used to assess habitual intake of fruits and vegetables. Individual carotenoid concentrations were determined for α-carotene (0.01 ± 0.05 µmol/L), β-carotene (0.17 ± 0.24 µmol/L), β-cryptoxanthin (0.07 ± 0.06 µmol/L), and lutein (0.06 ± 0.05 µmol/L). These results confirm the previously measured high prevalence of low serum vitamin A concentrations and adds information about low serum carotenoids among schoolchildren suggesting that they have low intakes of provitamin A-rich fruits and vegetables.

## 1. Introduction

Micronutrient deficiencies among children under 5 years old in low- and middle-income countries are common. Iron, vitamin A (VA), and zinc are specifically targeted for improvement by the World Health Organization (WHO) because their deficiencies are prevalent and lead to increased mortality and morbidity [1]. Community randomized controlled trials have shown that administering preformed VA solely or in combination with zinc to children in regions with a high prevalence of malaria, reduced morbidity [2,3]. Targeting preschool children is of specific interest because of long-term detrimental effects of undernutrition on cognitive development and adulthood work productivity [1]. VA deficiency (VAD) can lead to anemia, stunting, weakened resistance to infection, blindness, and death [1]. A random-effects meta-analysis of several VA trials showed reduction of mortality rates by 24% among children 6–59 months of age [4]. Current strategies to alleviate VAD are targeted high–dose VA supplementation to 6–59 months old children, fortification of foods that have high population coverage with preformed VA as retinyl palmitate and promoting the consumption of foods rich in VA and provitamin A carotenoids [1,5,6,7].

Despite a biannual campaign distributing high-dose VA capsules to children <59 months, VAD remains a public health problem in Burkina Faso [8]. Schoolchildren are assumed to be at lower risk but likely also suffer from micronutrient deficiencies in areas with low dietary diversity. Worldwide, 190 million preschool children are estimated to have VAD [1]. In Kaya (*n* = 214, age 8.5 ± 1.6 years) and Bogandé (*n* = 337, age 6.2–10.3 years), Burkina Faso, 47.2% and 37.1% of schoolchildren, respectively, had low serum retinol concentrations [9]. In Ouagadougou, 38.7% of 7–14 years old children attending private or public school had low serum retinol, which was more prevalent among children in public schools [10]. Low serum retinol concentration is an indicator of VAD and is currently recommended by WHO when used along with other biomarkers or surveys [11]. A prevalence of 20% of a given population group with serum retinol concentrations <0.7 μmol/L, is used to define VAD as a severe public health problem [11].

Micronutrient deficiencies in schoolchildren represent an additional concern because of increased nutritional needs for development, health, and academic performance [12]; this period can be as important as early infancy due to catch-up growth. In low- and middle-income countries where intake of animal-based foods is low, dietary provitamin A carotenoids are the main source of VA [13]. Due to economic reasons, limited knowledge and restricted access, the frequency and quantity of fruits and vegetables consumed by Burkinabe children is inadequate [7]. Increased access by children allowing repeat exposure showed an association with enhanced taste preferences towards increased fruit and vegetable intake [14]. Green leafy vegetables, orange fruits, and yellow-colored vegetables are rich in carotenoids and important for optimal health [13], and serum carotenoid concentrations reflect consumption of these fruits and vegetables [15,16]. The present study assessed serum retinol and carotenoid concentrations among schoolchildren (aged 7–12 years) in Burkina Faso to compare carotenoid-containing fruit and vegetable intake with other countries to determine relative consumption. 

## 2. Materials and Methods 

### 2.1. Study Area and Subjects

This study was conducted according to the guidelines laid down in the Declaration of Helsinki and all procedures involving human subjects/patients were approved by the Ethical Review Committee of Center MURAZ (001-2014/CE-CM). Written informed consent was obtained from parents or guardians of all included subjects. The trial was registered at Pan African Clinical Trials Registry (PACTR201702001947398). This cross-sectional study was conducted in the Western part of Burkina Faso, in primary school “A” of Kou’s Valley in Bama’s village in the Dande health district, between March and April 2014 during the dry season. Children attending the school were considered eligible if they were 7–12 years old living in the area; and their caregiver signed an informed consent form.

Exclusion criteria included clinical VAD, i.e., physiological ocular symptoms; severe anemia defined as hemoglobin (Hb) concentration <70 g/L [17]; serious illness (based on a medical examination) including tuberculosis and symptomatic human immunodeficiency virus infection. Children were enrolled based on the list of classes as a sampling frame. Recruitment covered the first four years of school (preparatory and elementary levels). We used *z*-score to assess anthropometric parameters based on WHO growth standards. Underweight was defined as WAZ more than 2 standard deviations (SDs) below the WHO median [18].

### 2.2. Data and Sample Collection

Venous blood (7 mL) was collected in EDTA tubes by trained nurses of Bama’s clinic and stored immediately on ice before transportation to the laboratories of the Institut de Recherche en Sciences de la Santé (IRSS, Bobo-Dioulasso). Vials were protected from light with aluminum foil to avoid photo-degradation of the carotenoids and retinol. Blood samples were centrifuged the same day at 3000 rpm for 10-min with a Universal 320R centrifuge (Hettich Zentrifugen, D-78532, Tuttlingen, Germany). Serum was transferred into brown 1.5 mL microcentrifuge tubes (Eppendorf, Hamburg, Germany) and stored at −20 °C until shipment to the University of Wisconsin–Madison, USA (UW) for serum retinol and carotenoid analyses. Malaria parasites were counted on thick blood smears prepared in the field from capillary blood. Hb concentration was measured on capillary blood with the 301+ Hemocue system (Angelholm, Sweden). Height to the nearest 0.1 cm and weight to the nearest 10 g were measured.

### 2.3. Serum Retinol and Carotenoid Extraction and Analys

Serum retinol and carotenoid analyses were performed at UW in the same analysis as described [15,16] with modifications. To 200–300 μL serum, ethanol (1.5 X v) containing 0.1% butylated hydroxytoluene (MP Biomedicals, Solon, OH, USA) as an antioxidant, and 25 μL C23-β-apo-carotenol as an internal standard were added followed by 3 extractions with hexanes (2.5 X v) (Fisher Scientific, Pittsburgh, PA, USA). Pooled hexanes were dried under nitrogen and reconstituted in 100 μL 50:50 (*v*/*v*) methanol:dichloroethane; 50 μL was injected into a Waters HPLC [16] equipped with a Sunfire C18 (5 μm, 4.6×250 mm) analytic column (Waters, Inc., Milford, MA, USA) and a guard column. Samples were run at 1 mL/min using 70:30 (*v*/*v*) methanol:water (solvent A) and 80:20 (*v*/*v*) methanol:dichloroethane (solvent B), both with 10 mM ammonium acetate as a modifier, with the following gradient: 10-min linear gradient from 50% A:50% B to 100% B, followed by a 20-min hold, 3-min linear gradient to 50% A:50% B, and 6-min equilibration. Chromatograms were evaluated at 450 nm for carotenoids and 325 nm for retinol using purified external standards.

### 2.4. Statistical Methods 

All analyses were conducted using the Statistical Analysis System (version 9.4; SAS Institute, Cary, NC, USA). Descriptive statistics were used to summarize nutritional characteristics of the participants as well as the main outcomes. *z*-scores were determined using WHO Anthro. The difference between Body Mass Index (BMI)-for-age *z*-scores of the children and WHO standards (*z*-score = 0) was analyzed by one-sample Student’s *t*-test. Data were reported as mean ± SD.

## 3. Results

### 3.1. Subject Characteristics

**Figure 1:**
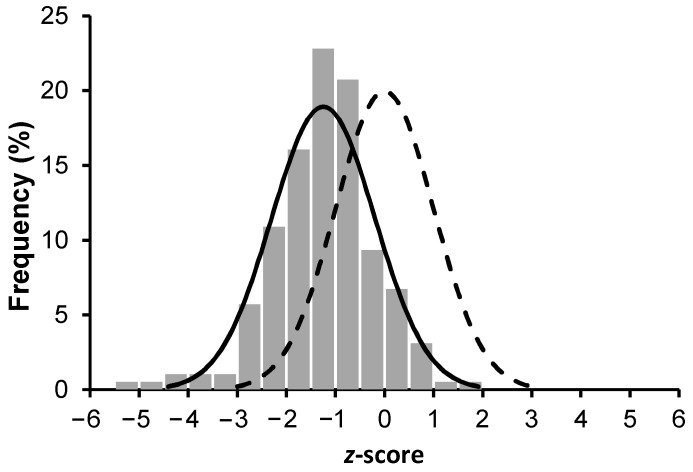
BMI-for-age *z*-score distribution in children aged 7–12 years (*n* = 193) in Burkina Faso. The normal distribution curves are given for these children (solid line, mean = −1.24 ± 1.05) compared with the WHO child growth standards for children 61 months–19 years of age (dashed line, mean = 0 ± 1).

All children (*n* = 193) met inclusion criteria and none of them had ocular signs of VAD, had blood samples drawn, and had anthropometric parameters measured (Table 1). Comparing BMI-for-age *z*-score (BAZ) of the children to WHO growth standards determined that mean BAZ was −1.24 ± 1.05 (different from 0, *p* < 0.0001) with 20% (CI = 95%) low BAZ (Figure 1). The children had high prevalence of underweight (17.6% with CI = 95%), stunting (25.2% with CI = 95%), asymptomatic malaria (39.5%), and prevalent anemia (23% with Hb concentration < 110 g/L).

### 3.2. Serum Retinol and Carotenoid Concentrations

Mean serum retinol concentration was 0.80 ± 0.35 µmol/L with 46% (88 children out of 193) <0.7 µmol/L (the cut-off for VAD) [11], although the utility of this measurement is limited as markers of chronic or acute inflammation (e.g., α_1_-acid glycoprotein and C-reactive protein) were not taken to correct for the impact of infection in these children, which is known to lower serum retinol independent of VA status [11]. Concentrations of lutein, α- and β-carotene, and β-cryptoxanthin were determined and compared with other studies in children (Table 2). Total serum carotenoids ranged from 0 to 2.82 µmol/L with a mean of 0.23 ± 0.29 µmol/L. Serum carotenes had a low mean concentration (0.13 ± 0.24 µmol/L) well under the reference range (0.9–3.7 µmol/L) [16], and only two children were within this reference range. Serum retinol concentrations were not related to serum provitamin A carotenoid concentrations *p* > 0.05).

## 4. Discussion

The high prevalence of low serum retinol concentrations is consistent with research in other regions from Burkina Faso [9,10]. One-quarter of the children were anemic, with almost 40% of those presenting with asymptomatic malaria. Serum concentrations of the four measured carotenoids were lower than those found in other populations, including young preschool children and schoolchildren from other low-income countries (Table 2) [16,19,20,21,22]. Low fruit and vegetable consumption has been recognized as a key contributor to inadequate micronutrient intake and deficiencies affecting optimal health [13]. Intake of provitamin A-source foods was likely quite low as demonstrated by low serum retinol and carotenoid concentrations. Previous studies in Bolivia, Burkina Faso, and the Philippines showed that the diets are mainly based on cereals and tubers with white flesh and poor in carotenoids [23,24]. 

Staple foods are traditionally consumed with vegetable sauces in Burkina Faso, which could be good carotenoid sources, but data from previous studies showed high intake of vegetables that are poor in carotenoids, such as dried okra. The Institute of Medicine has defined bioefficacy of dietary β-carotene, α-carotene, and β-cryptoxanthin as 12, 24, and 24 µg to 1 µg retinol activity equivalents (RAE), respectively [25]. Dried okra, which has <5 μg RAE/100 g [26], is reportedly consumed daily in rural areas of Burkina Faso [24]. On the other hand, sorrel (*Rumex acetosa* L., a small green herb), which contains 75–140 μg RAE/100 g [26,27], is reportedly consumed in only one meal in six days (generally market day) [26,27] while Weber and Grune showed that carotenoids make up 35% of total VA intake in industrialized countries [28].

Dietary data collected previously showed that intake of fruits and vegetables is generally poor, due to low frequency and small quantities [26,27]. An intervention was able to increase intake of sorrel leaves and provide an extra 35–49 μg RAE to schoolchildren (Table 3) [27]. A previous 4-month trial in Burkina Faso showed that red palm oil (RPO), naturally rich in carotenoids, can cover daily VA needs for children <6 years of age [10]. Rural populations cannot afford refined VA-fortified oil, so promotion of RPO may be appropriate and add VA-value to vegetables poor in RAE, such as okra [26,27]. Although, the study village produced agricultural products, dietary habits, and cultural restrictions limited food diversification. Most people do not consume papaya for cultural reasons [26]. Other fruits and vegetables constitute an income for mothers who sell the products instead of using them for household consumption [26]. 

Serum carotenoid concentrations are influenced by bioavailability and overall bioefficacy [13,25]. Bioconversion of provitamin A carotenoids to retinol is affected by VA status and host-related facts, such as genetic polymorphisms [25]. Because different bioefficacy factors are used for provitamin A carotenoids, food composition data tables should report food VA content in mass of each carotenoid whenever possible [13]. We recognize some shortcomings of the study. The lack of dietary data on intake of fruits, vegetables, and other sources of VA and carotenoids is an important limitation of our study. Furthermore, biomarkers of inflammation like C-reactive protein and α_1_-acid glycoprotein, which impact serum retinol, were not measured [11]. Malaria in Burkina Faso is endemic, which has been associated with low serum retinol concentrations in Thailand [29]. Serum retinol also suffers from sensitivity and specificity issues [11]. Determination of confounding factors and the causes of low serum retinol concentrations in this community would allow us to form more concrete associations among variables. 

This study biochemically suggested poor fruit and vegetable intake among schoolchildren in Burkina Faso and demonstrated that low serum retinol concentrations are still prevalent, adding to the body of evidence that large-scale promotion of naturally rich RPO or food fortification may be needed among the population. Vegetable intervention studies should be pursued to evaluate whether locally available vegetables could be efficacious. Additionally, it shows the importance of behavior change education to increase dietary diversity. Dietary validation studies of self-reported fruit and vegetable intake should ideally include measurement of serum biomarkers of intake. Fruits and vegetables should be available and accessible both at home and at school. To date, there are a limited number of studies evaluating school-based environments to increase availability and accessibility of fruits and vegetables for children.

## Figures and Tables

**Table 1:** Subject characteristics.

Parameter	Value (mean ± SD)
Age, year	9.3 ± 1.48
Height, cm	129.1 ± 12.4
Weight, kg	24.8 ± 5.37
Height-for-age *z*-score	−0.73 ± 1.25
Weight-for-age *z*-score	−1.16 ± 1.01
BMI-for-age z-score	−1.24 ± 1.05
Hemoglobin, g/L	122.4 ± 10.9
Positive malaria blood smear, % (average parasitemia)	39.5 (1655 parasites)

**Table 2:** Serum carotenoids concentrations (±SD) or range in apparently healthy children from various regions of the world.

Country	Age Years	*n*	Retinol µmol/L	β-Carotene µmol/L	α-Carotene µmol/L	β-Cryptoxanthin µmol/L	Lutein µmol/L	Reference
**Burkina Faso**	7–12	193	0.80 ± 0.35	0.12 ± 0.21	0.01 ± 0.04	0.05 ± 0.06	0.05 ± 0.06	Present study
**(range)**			(0.17–1.92)	(0.004–2.15)	(0–0.4)	(0–0.3)	(0–0.2)	
**Zambia**	5–7	123	0.98	0.76	0.62	0.10	0.86	Mondloch et al. [16]
**Senegal**	2–4	281	-	0.16	0.030	0.020	0.46	Rankins et al. [19]
**India**	2–11	50	1.10	0.31	0.035	0.12	0.42	Das et al. [20]
**Philippines**	9–12	27	0.87	0.23	0.03	0.07	0.23	Ribaya-Mercado et al. [21]
**USA**	6–7	839	-	0.34	0.075	0.21	0.34 ^a^	Ford et al. [22]

^a^ This value includes the xanthophyll zeaxanthin.

**Table 3:** Retinol activity equivalents (RAE) from sauce intake in Burkinabe children and coverage of the child’s vitamin A requirements in relationship to the Estimated Average Requirements and the Recommended Daily Allowances.

Characteristic	Normal Diet ^a^		Added Green Leafy Vegetables ^b^	
Age group	6–9 years	≥10 years	6–9 years	≥10 years
Number of meals/day	2	2	2	2
Amount of sauce ingested/day (g)	184	254	184	254
Mean retinol activity equivalents (μg RAE/100 g)	36	36	55	55
Mean intakes of retinol activity (μg RAE/day)	66	91	101	140
Estimated average requirements [25] (μg RAE/day)				
Boys (6–13 years)	275–445	445	275–445	445
Girls (6–13 years)	275–420	420	275–420	420
Recommended Daily Allowance [25] (g RAE/day)	400–600	600	400–600	600
% of estimated average requirements met/day for boys and girls aged 6–13 years	15–24	20–22	23–37	31–33

Data based on information in reference [26]; RAE, Retinol Activity Equivalent. ^a^ The normal diet included 60 households in a rural area of Burkina Faso that received no intervention. ^b^ Green leafy vegetables were provided to 30 households from the same area that received locally-produced sorrel leaves at intervals corresponding to market day frequencies (every 3 days) for two months.
